# The impact of new population policies on women's reproductive health pattern in Ardabil, Iran: a comparative cross-sectional study

**DOI:** 10.3389/frph.2025.1520027

**Published:** 2025-04-11

**Authors:** Nazila Nejhaddadgar, Afrouz Mardi, Maryam Zare

**Affiliations:** ^1^ Public Health Department, Health Faculty, Ardabil University of Medical Sciences, Ardabil, Iran; ^2^ School of Medical Sciences, Khalkhal Faculty of Medical Sciences, Ardabil, Iran

**Keywords:** new population policies, reproductive health, women, Ardabil, Iran

## Abstract

**Background:**

Family planning helps couples make voluntary, informed, and responsible decisions about having children based on their economic, physical, and psychological capabilities. New population policies have banned family planning services in Iran. This study sought to investigate the impact of these policies on women's reproductive health pattern in Ardabil, Iran.

**Methods:**

A comparative cross-sectional study was conducted on 800 eligible women (in two groups of 400, pre/post new policies) attending health centers in Ardabil. Health centers were chosen using a stratified cluster method, and the eligible women were selected through convenience sampling. A researcher-designed questionnaire was used to collect data on demographic characteristics, reproductive health history, and contraceptive use in the past six months. Data were analyzed using SPSS version 24, with Z and Chi2 statistical tests.

**Results:**

The majority of women were aged 15–24 (45.25% and 53%), had education levels below a diploma (52.5% and 47%), and were housewives (88.5% and 84.75%). Prior to the policy change, 67.25% of women used modern contraception methods, compared to 31% after. Rates of unwanted pregnancies increased from 28.5% to 49.25%, and the incidence of one abortion rose from 14.25% to 21.75%. These differences were statistically significant (*p* < 0.05).

**Conclusion:**

The ban on family planning services has led to a shift towards traditional contraception methods, an increase in unwanted pregnancies, and unsafe abortions. This highlights the need for health policymakers to implement appropriate interventions.

## Background

One of the important population developments in the last three decades in Iran has been the rapid decline in fertility (1). In Iran, before the Islamic Revolution, family planning policies were considered by governmental officials as the basis for development and had been implemented in a decentralized manner since 1955. With the implementation of family planning policies in the decade of 1966–1975, the population growth rate decreased slightly from 3.1 to 2.7 percent. After the Islamic Revolution, executive policies were directed towards encouraging population growth. After the end of the imposed war, the population control policy was re-approved and implemented. The continuation of this policy had consequences whose harmful effects were revealed in the 1991 census, namely, the fertility rate dropped to 1.8, which is less than the population replacement rate (1.2) (2). It is expected that by the end of this century, Iran will witness a negative growth in population. Currently, this decrease continues, with the rate now at 1.62 children. This change has raised significant concerns for policymakers (1, 3). In response to this rapid decline in fertility, the Supreme Leader announced new population policies in April 2014 (4).

The new population policies aim to increase the population and fertility rate (5). These policies goals are: removing obstacles to marriage, financially supporting young couples and empowering them to meet living expenses, paving the way for reducing the average age of marriage, improving family livelihoods, ensuring health and healthy nutrition, promoting easy and stable marriages, and preventing divorce by encouraging peace and reconciliation between couples, providing insurance coverage for childbirth expenses, infertility treatment, teaching life and communication skills, and providing counseling services, allocating appropriate facilities for mothers, especially during pregnancy, breastfeeding, student life, employment, and so on (6).

They have gradually limited the provision of family planning services over the years. At first, permanent prevention, methods like vasectomy and tubectomy were legally condemned. Finally, on January 30, 2022, was implemented a complete ban on access to contraceptives. This ban prohibits the any provision of contraceptive methods, even encouraging their use in health care centers. While before 2022, non-surgical contraceptives were still provided in a limited way (5).

Declining population growth is a general problem in developed countries as well. A multi-country study which was conducted with 19 countries, stated that there are strategies that support childbearing without limiting access to contraception, like re-employment after childbearing, women's current employment, availability of daycare, and flexible work options, employment support, access to fertility services or income support, infertility treatment and reproductive care, tax concession for each baby, universal payments, stay-at-home mums, child care and early childhood education, parental flexible hours of work, employment policies, and so on. The fact that these strategies increase fertility suggests that there are policies that support reproductive autonomy and increase fertility (7–9).

conversely, policies that restrict autonomy, like restricting access to family planning services, have negative health impacts.Turkey currently pursues an aggressive pronatalist population policy by restricting access to abortion services (10). While, China replaced its one-child policy in 1979, with the universal two-child policy from 2015 and three-child policy from 2021 (11).

These policies are different depending on the culture and social status of the countries. The strong prenatal orientation of sovereignty creates a situation of reproductive injustice that may threaten the health of mothers (11). The role of family planning programs in improving mother and child health is undeniable. The health benefits of fertility with appropriate planning and at the right age, which is one of the goals of family planning programs,uncontroversial (5). Family planning helps couples make voluntary, informed, and responsible decisions about having children based on their economic, physical, and psychological capabilities and balance between sustainable development and population growth (12).

Reports have indicated health consequences of changing the family planning approach, such as unwanted pregnancies, an increase in unsafe abortions, and maternal and child mortality in vulnerable groups (13–15).

The average fertility rate of Ardabil province in 2019 was about 1.8%, but in 2022 it decreased to 1.6%, which is lower than the national average (16).

Reproductive health encompasses all critical stages in ensuring the health of family members, especially women and girls, from birth to death. Expanding reproductive health and addressing its various dimensions at the national and international level is crucial for ensuring the health of society and family with a focus on women's health (12, 17, 18). Reducing maternal and child mortality, preventing fatal diseases, and increasing the quality of life are direct results of reproductive health programs. According to the action plan and document of the International Conference on Population and Development in 1994 in Cairo, reproductive health is defined as; complete physical, mental and social well-being, related to the reproductive system. Reproductive health means that individuals have the ability to reproduce freely and consciously, making their own decisions about how and when having children. Access to reliable, effective, low-cost and acceptable family planning methods, as well as access to health and medical services for safe pregnancy and childbirth, are essential components of reproductive health (12).

Studies have shown that restrictions on family planning services can lead to adverse health consequences, especially for vulnerable sections of society (13, 19). Despite these findings, few studies have investigated the patterns of women's contraception methods and reproductive health before and after the implementation of new fertility promotion policies. In Ardabil city, as in other regions, there has been a significant decrease in fertility in recent years. Therefore, the main objective of this study was to determine the impact of new population policies on the reproductive health pattern of women in Ardabil, Iran.

## Methods

### Data

A cross-sectional comparative study was conducted in two periods: first, in the summer of 2021, before the contraception ban, and second, in the summer of 2024, more than two years after contraception ban. The study aimed to enroll a total of 800 participants who were referred to health centers affiliated with Ardabil University of Medical Sciences in Ardabil city. (400 in the period 1, before the contraception ban and 400 in the period 2, following the contraception ban.) This sample size allowed for (*P* = 50%, 95% confidence coefficient and 5% accuracy).It was initially set at 383 people. However, to increase the accuracy of the results, 400 people were included in each period.

It is important to note that the Director General of Medicines and Materials Affairs of the Ministry of Health of Iran issued an executive order prohibiting access to any contraceptives in health centers on January 30, 2022 (20). Ardabil Province is situated in northwestern Iran and has an estimated population of 1,300,000 people, the majority of whom are Muslims. It covers an area of approximately 18,000 km^2^ or about 1% of Iran's total area (21, 22). Ardabil city has 34 health care centers. Each center provides a wide range of services, including medicine, dentistry, maternal and child health, family planning, mental health, environmental health, and so on. The family planning department has been practically deactivated after the implementation of the new policies, and only follows policies to encourage childbearing, so the users of contraceptives have been excluded.

The inclusion criteria for the study were married women under 49 years of age, non-pregnant, with at least one pregnancy history, living in Ardabil city. The exclusion criteria were having an addiction and incomplete completion of the questionnaire.

Stratified cluster sampling was used to select health care centers from five city regions (north, south, center, east, and west), and two health care centers randomly chosen from each region (totally 10 centers) in both periods. In the period 1, the research samples were randomly selected among the medical records of eligible women and the researcher filled the questionnaires. A phone call was made if more information is needed. In the period 2, the eligible women who referred to the centers, were recruited through convenience sampling and the questionnaires were completed by the researcher through interviews. The center staff collaborated with the researcher in all these steps.

A researcher-made questionnaire was used as the data collection tool, covering demographic characteristics, reproductive health history, and contraceptive methods used in the last six months.

Reproductive variables including; the number of pregnancies, age of first pregnancy, unwanted pregnancy, abortion, type of contraceptive, method of childbirth, and stillbirth. Contraceptives such as pills, injections, IUDs (Intra Uterine Devices), tubectomy, vasectomy, and condoms are considered modern methods, while, withdrawal, breastfeeding, and rhythmic contraception are considered traditional methods.

Additional information, if needed, was obtained through telephone calls or was extracted from the integrated health system. For example, some of the questions include: Have you had an unwanted pregnancy in the last year? Have you had an elective abortion in the last year? What kind of contraceptives have you used in the last six months?

The data was analyzed using SPSS version 24 software, with *Z* and *χ*^2^ statistical tests and multivariable logistic regression. *P* value less than 0.05 was considered significant.

### Ethics statement

This study was the result of a research project with ethics approval from the Ethics Committee of the Research Deputy in Ardabil University of Medical Science (IR.ARUMS.REC.1401.051). All information is confidential and cannot be used to identify the respondents.

## Results

Table 1 shows the demographic characteristics of participants in the two time periods. The highest percentage in both groups belonged to the age group of 15–24 years (45.25% and 53%) and then 25–34 years (33.5% and 29.75%). The majority of women in both education groups had below a diploma (52.5% and 47%) and were housekeepers (88.5% and 84.75%).

**Table 1:** Demographic characteristics of women participating in the study in the two time periods.

Year	2021 (period 1)	2024 (period 2)
	*N* (%)	*N* (%)
Demographic characteristics	400 (100)	400 (100)
Age		
15>	3 (0.75)	5 (1.25)
15–24	181 (45.25)	212 (53)
25–34	134 (33.5)	119 (29.75)
35–44	71 (17.75)	51 (12.75)
45–49	11 (2.75)	13 (3.25)
Mean (SD)	28.12 (±3.68)	28.66 (±2.44)
Education		
Elementary	45 (11.25)	47 (11.75)
Undergraduate	210 (52.5)	188 (47)
Diploma and postgraduate diploma	126 (31.5)	140 (35)
Bachelor's degree	15 (3.75)	19 (4.75)
Masters and PhD	4 (1)	6 (1.5)
Occupation		
Housewife	354 (88.5)	339 (84.75)
Employed	46 (11.5)	61 (15.25)
Spouse education		
Elementary	50 (12.5)	47 (11.75)
Undergraduate	195 (48.75)	188 (47)
Diploma and postgraduate diploma	78 (19.5)	140 (35)
Bachelor's degree	65 (16.25)	19 (4.75)
Masters and PhD	12 (3)	6 (1.5)
Spouse occupation		
Unemployed	44 (11)	38 (9.5)
Official employee	116 (29)	139 (34.75)
Self-employed	240 (60)	223 (55.75)
Socioeconomic status		
Very poor	203 (50.75)	56 (14)
Poor	182 (45.5)	254 (63.5)
Middle	12 (3)	65 (16.25)
Rich	3 (0.75)	25 (6.25)

Differences in fertility and contraception use between the two periods is shown in Table 2. So that, 67.25% of women used modern methods of contraception in period 1, which decreased to 31% in period 2. The percentage of women not using any contraceptives increased from 13% to 25.5%. This difference was statistically significant (*P* = 0.001). Additionally, the study showed that 49.25% of women experienced an unwanted pregnancy in period 1, compared to 28.5% in period 2. This increase was also statistically significant (*P* = 0.001). The abortion rate rose from 14.25% before contraception ban to 21.75% after that. The percentage of women with a history of two or more abortions increased from 0.75% to 4%, which was statistically significant (*P* = 0.011). Multi-variable logistic regression analysis shows that as the number of unwanted pregnancies increases, the risk of abortion also increases. It also suggests that the number of unintended pregnancies and abortions increased in groups with a higher number of pregnancies, while the number of stillbirths and other complications did not show clear results. There was no statistically significant difference in the variables of age of first pregnancy, number of pregnancies, number of stillbirths, and method of termination of pregnancy when comparing the two periods before and after contraception ban.

**Table 2:** Multivariable logistic regression model for the association between contraception ban (in the two time periods) and women's reproductive health variables.

			Odds ratio			
Year	2021 (period 1)	2024 (period 2)	Crude (95% CI)	*P*-value	Adjusted (95% CI)	*P*-value
	*N* (%)	*N* (%)				
Variables	400 (100)	400 (100)				
Age categories						
15>	8 (2)	12 (3)	1	–	1	–
15–24	269 (67.25)	299 (74.75)	1.04 (0.86–1.56)	0.102	1.06 (0.87–1.48)	0.220
25–34	113 (28.25)	80 (20)	1.21 (0.90–1.85)	0.089	1.36 (0.96–1.79)	0.091
35–44	9 (2.25)	7 (1.75)	1.95 (0.99–2.71)	0.061	0.22 (0.08–1.05)	0.024
45–49	1 (0.25)	2 (0.5)	2.16 (0.98–3.31)	0.106	0.58 (0.38–1.13)	0.146
Contraceptive methods						
No method (ref.)	52 (13)	102 (25.5)	1	–	1	–
Traditional methods	79 (19.75)	174 (43.5)	0.72 (0.64–0.82)	<0.001	0.66 (0.59–0.81)	<0.001
Modern	269 (67.25)	124 (31)	0.71 (0.40–0.84)	<0.001	0.66 (0.58–0.82)	<0.001
Gravida						
1 (ref.)	273 (68.25)	250 (62.5)	1	–	1	–
2	112 (28)	121 (30.25)	1.32 (0.89–1.46)	0.090	0.83 (0.48–1.46)	0.131
3≤	15 (3.75)	29 (7.25)	1.49 (0.93–1.67)	0.101	0.58 (0.38–1.13)	0.220
Number of unwanted pregnancies						
0 (ref.)	286 (71.5)	196 (49)	1	–	1	–
1	114 (28.5)	197 (49.25)	0.85 (0.71–0.95)	<0.001	0.81 (0.71–0.91)	<0.001
2≤	0 (0)	7 (1.75)	0.50 (0.40–0.64)	<0.001	0.73 (0.63–0.84)	<0.001
Number of abortion						
0 (ref.)	340 (85)	297 (74.25)	1	–	1	–
1	57 (14.25)	87 (21.75)	0.89 (0.53–1.48)	<0.001	0.85 (0.71–0.95)	<0.001
2≤	3 (0.75)	16 (4)	1.71 (1.13–2.78)	<0.001	1.64 (1.15–1.94)	<0.001
Number of stillbirth						
0 (ref.)	388 (97)	390 (97.5)	1	–	1	–
1	10 (2.5)	7 (1.75)	1.01 (0.90–1.14)	0.332	0.97 (0.85–1.10)	0.680
2≤	2 (0.5)	3 (0.75)	0.58 (0.38–1.13)	0.075	0.60 (0.49–1.05)	0.068
Type of last delivery						
Natural	188 (47)	175 (43.75)	0.28 (0.11–1.15)	0.377	0.22 (0.08–1.05)	0.302
Cesarean section	212 (53)	225 (56.25)	0.60 (0.49–1.05)	0.714	0.89 (0.53–1.48)	0.615

## Discussion

The results of this study showed that, after the implementation of the new policies, the majority of women shifted to traditional contraceptive methods, the number of women not using any contraceptive doubled and the odds of experiencing an unwanted pregnancy or abortion increased, which are associated with poorer maternal health outcomes.

This study add to a growing evidence that restricting access to modern contraceptive methods is associated with an increase in use of unsafe contraceptive methods and abortion. Khalajabadi (2016) conducted a study in Sanandaj before the prohibition law was implemented and reported a similar shift from safe to traditional and unsafe contraceptive methods. They suggested that this change was influenced by increased awareness and education among women and men as well as preference for non-hormonal methods with fewer side effects. They also reported that, the level of access, quality, promotion and information about contraceptives has decreased. Also, government health centers have been the most important source of contraceptives for rural, low-educated, low-income, and housewives women (13).

Another significant finding of our study was the increase in unwanted pregnancies after the new policies. Maghalian et al. found that nearly one-third of pregnancies in Tabriz were unwanted in the first year of implementation of new policies with unwanted pregnancies predicting poorer pregnancy outcomes (23). Zendehdel and Jahanfar also reported a relatively high rate of unwanted pregnancies among married women (44.9%) in the south of Tehran. They believe that people with low financial power and low education may not be able to pay for contraceptive methods and may not seek the necessary information about contraception (24).

Additionally, the results of our study showed that the rate of abortion in women of reproductive age has increased significantly after the implementation of the new policies, which is not surprising given the increase in unwanted pregnancies. Numerous studies have suggested that restricting access to contraception is associated with increases in unwanted pregnancy and abortion (13, 25, 26). For instance, Shirdel et al. concluded that the new population policies in Iran, including restricting access to contraception, prenatal screening and abortion, may lead to an increase in unsafe abortion and its consequences (26).

Critics of Iran's new demographic policies believe that limited access to contraceptives increases the number of unwanted pregnancies and unsafe abortions (25).The World Health Organization defines unsafe abortion as the termination of pregnancy by any method, by people lacking appropriate skills and/or in an unsanitary and non-medical environment (27).

Therefore, the results of this study confirm that restrictions on family planning services can lead to adverse health outcomes for mothers and children. In simple terms, when most couples prefer to have one or two children, restrictions on family planning services not only do not increase the fertility rate, but also lead to an increase in the rate of abortion and its related consequences (25).

## Limitations

One limitation of the present study is that the questionnaire was self-reported and sometimes completed by telephone, which may be associated with non-reporting or misreporting of some questions. Another limitation was that due to the illegality of elective abortion in Iran, the actual abortion rate may be higher than the reported rate. And that, this study design does not establish causal relationships but shows an important association.

## Conclusions

It appears that the new population policy in Iran needs to be reevaluated. The strict laws and the lack of access to family planning services may be contributing to an increase in the use of traditional methods of contraception. This, in turn, could be leading to an increase in unwanted pregnancies and unsafe abortions, both of which are significant issues. These issues clearly pose a threat to the health of both mothers and children. Further research is also recommended on the impact of new policies on women's reproductive health patterns, especially qualitative research, which should be conducted separately in urban and rural areas.

## Data Availability

The raw data supporting the conclusions of this article will be made available by the authors, without undue reservation.
